# Emerging role of free triiodothyronine in patients with anti-N-methyl-D-aspartate receptor encephalitis

**DOI:** 10.1038/s41598-021-85596-6

**Published:** 2021-03-15

**Authors:** Tuo Ji, Zhi Huang, Yajun Lian, Chengze Wang, Qiaoman Zhang

**Affiliations:** Department of Neurology, Zhengzhou University First Affiliated Hospital, Zhengzhou city, 450052 Henan Province China

**Keywords:** Central nervous system infections, Neurology, Encephalopathy

## Abstract

We aimed to investigate the role of free triiodothyronine (FT3) in patients with anti-N-methyl-D-aspartate receptor (anti-NMDAR) encephalitis. 137 consecutive inpatients (2016–2019) were registered prospectively and followed up for 12 months. 96 eligible patients were included in the study. The modified Rankin scale (mRS) score was collected, and the score of 3–6 was defined as a poor outcome. The patients were equally classified into 3 subgroups based on their FT3 levels obtained within 24 h of admission, and the subgroup differences were analyzed by parametric or nonparametric tests as appropriate. Logistic regression analysis was performed. We found that there was no difference in the mRS scores upon admission among 3 subgroups, however, patients in the low-FT3 subgroup tended to have higher disease severity during hospitalization and worse outcome in follow-up visits, represented by higher chances of intense care unit (ICU) admission (*P* < 0.001), longer hospital stay (*P* < 0.001), greater maximum mRS scores during hospitalization (*P* = 0.011), lower rates of getting clinical improvement within 4 weeks of starting treatment (*P* = 0.006), and higher percentages of poor 1-year outcome (*P* = 0.002). The level of FT3 was an independent factor correlated with ICU admission (*P* = 0.002) and might be a potential predictor for 1-year outcome. Our preliminary results suggest that the FT3 may be a risk factor involved in the evolution and progression of anti-NMDAR encephalitis, whereas the underline mechanisms remain to be explored. Attention should be paid to these patients with relatively low FT3 upon admission, which might possibly aid clinical prediction and guide clinical decision-making.

## Introduction

Anti-N-methyl-D-aspartate receptor (anti-NMDAR) encephalitis is a newly identified autoimmune encephalitis associated with antibodies against functional NMDA receptors that predominantly affects young females and exhibits a well-defined set of clinical features^1^. To tackle the challenges of early diagnosis and risk stratification, several clinical prognostic factors have been developed. Altered consciousness, intensive care unit (ICU) admission and no use of immunotherapy seem to be variables associated with poor outcome in anti-NMDAR encephalitis, whereas other factors, including age, gender, abnormalities of cerebrospinal fluid (CSF) and changes of magnetic resonance imaging (MRI), remain controversial^2,3^. The “anti-NMDAR Encephalitis One-Year Functional Status (NEOS) score”, proposed by Balu and his colleagues, contained 5 variables such as ICU admission, treatment delay > 4 weeks, lack of clinical improvement within 4 weeks of starting treatment, abnormal MRI, and CSF white blood cell (WBC) count > 20 cells/μL. Each of them was assigned 1 point to create the NEOS score, which was strongly associated with the probability of poor functional status at 1 year^4^. It seemed to be a reliable tool to predict the 1-year outcome and was recently validated by another team in Chinese population^5^. However, some of the variables may not be obtained until 4 weeks of starting treatment, which in some extent limit its use in the early stage of the disease. There is urgent need for novel prognostic biomarkers, especially those that can be obtained early, which could help estimate the velocity of clinical progression in advance.

Abnormal serum levels of thyroid hormones have been reported in patients with a variety of non-thyroidal illnesses. The free triiodothyronine (FT3), an indicator of thyroid function, has been proven to be associated with a worse cardiovascular risk factor profile and could lead to progression of atherosclerosis^6^. Besides that, emerging evidence suggests that the FT3 may also serve as a prognostic factor in several critical diseases (e.g., sepsis, respiratory failure, and stroke) and some autoimmune diseases (e.g., systemic lupus erythematosus and neuromyelitis optica spectrum disorders)^7–12^. In clinical practice of treating anti-NMDAR encephalitis, we noticed that several patients with relatively low FT3 upon admission deteriorated fast and achieved an unfavorable outcome eventually. Whether this is an accidental event or there are some underline correlations is of interest to investigate. To date, there are only a few data on this issue^13,14^, and the evidence gathered to date is inconclusive. Herein, we aimed to investigate the role of the FT3 in patients with anti-NMDAR encephalitis, from the perspectives of clinical picture during hospitalization and outcome in 1-year follow-up visits.

## Results

### Descriptive characteristics of the study population

**Figure 1:**
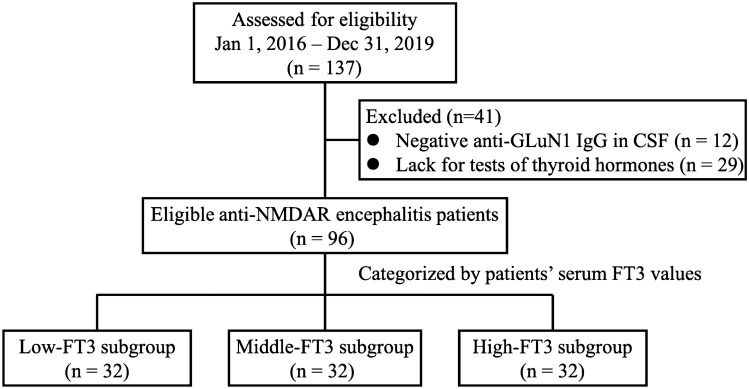
Cohort selection process of the study population. Of 137 admissions for anti-NMDAR encephalitis reviewed, 12 patients were excluded because their IgG anti-GluN1 antibodies were only positive in serum but negative in CSF, which couldn’t meet the diagnostic criteria of definite anti-NMDAR encephalitis. Another 29 patients were excluded for the lack of results of thyroid hormones within 24 h of admission. Ultimately, a total of 96 patients were included in this study. Based on the levels of serum FT3, the patients were equally divided into three subgroups, i.e. the low-FT3, middle-FT3 and high-FT3 subgroup.

In total, 137 consecutive patients were screened, and 96 patients met the inclusion criteria (median 30 [21–40.5] years; 44.79% females). Figure 1 summarizes the cohort selection process, and the baseline clinical features of the study population is shown in Table 1. The most common main symptoms included psychiatric behaviors (79.17%), seizures (65.63%) and cognitive dysfunction (58.33%). More than half of the patients had abnormal MRI (57.29%) and pneumonia (56.25%), but only a few of them (5.21%) were detected with tumors, including 2 pulmonary cancers and 3 immature ovarian teratomas. A quarter of the patients had thyroid dysfunction, including 11 patients with subclinical hyperthyroidism (11.46%), 1 patient with clinical hyperthyroidism (1.04%), 2 patients with subclinical hypothyroidism (2.08%), 6 patients with low T3 syndrome (6.25%), 4 patients with other conditions (4.17%). A majority of the patients (94.79%) received first-line immunotherapies, but only a minority of them (12.5%) were treated with second-line immunotherapies additionally. All patients were followed for 12 months, and most of them (76.04%) achieved a favorable outcome. A small number of the patients (6.25%) died within 12 months due to serious complications, including 3 patients with severe infections and 3 patients with intractable status epileptics.

**Table 1:** Clinical features and subgroup comparisons of the study population.

	Total	Low-FT3 subgroup	Middle-FT3 subgroup	High-FT3 subgroup	*P*
**Data obtained within 24 h of admission**					
Cases, n	96	32	32	32	
Females, n (%)	43 (44.79)	18 (56.25)	11 (34.38)	14 (43.75)	0.235
Age at onset, median (IQR), years	30 (21–40.5)	34 (23.5–48.5)	29.5 (23–39.5)	26 (17–33)	0.004*
The mRS score upon admission, mean (SD)	2.69 (0.79)	2.72 (0.68)	2.69 (0.93)	2.66 (0.79)	0.953
ICU admission, n (%)	16 (16.67)	6 (18.75)	6 (18.75)	4 (12.5)	0.834
Symptoms within 24 h of admission					
Prodromal symptoms, n (%)	61 (63.54)	22 (68.75)	20 (62.50)	19 (59.38)	0.804
Psychiatric behavior, n (%)	72 (75)	27 (84.38)	23 (71.88)	22 (68.75)	0.329
Cognitive dysfunction, n (%)	56 (58.33)	16 (50)	22 (68.75)	18 (56.25)	0.351
Speech dysfunction, n (%)	41 (42.71)	12 (37.5)	14 (43.75)	15 (46.88)	0.813
Seizures, n (%)	55 (57.29)	17 (53.12)	18 (56.25)	20 (62.5)	0.813
Movement disorder, n (%)	35 (36.46)	12 (37.5)	13 (40.63)	10 (31.26)	0.804
Consciousness declination, n (%)	20 (20.83)	9 (28.12)	6 (18.75)	5 (15.62)	0.540
Autonomic dysfunction, n (%)	33 (34.38)	15 (46.88)	11 (34.38)	7 (21.88)	0.123
Central hypoventilation, n (%)	10 (10.42)	5 (15.62)	3 (9.38)	2 (6.25)	0.369
Blood tests					
WBC count, median (IQR), n × 10^9^/L	8.5 (6.6–11.05)	9.15 (7.45–12.8)	8.75 (7.11–10.8)	6.99 (6.2–8.9)	0.047*
CRP, median (IQR), mg/L	2.1 (0.98–7.21)	6.87 (2.21–11.11)	1.69 (0.74–3.02)	1 (0.17–2.2)	< 0.001*
Thyroid status					
FT3, median (IQR), pmol/L	4.45 (3.88–5.01)	3.66 (3.37–3.88)	4.45 (4.36–4.62)	5.31 (5.01–5.59)	–
FT4, median (IQR), pmol/L	11.97 (10.63–13.52)	12.37 (9.89–13.33)	11.78 (10.84–13.21)	12.03 (10.82–14.69)	0.966
TSH, median (IQR), uIU/ml	1.07 (0.59–1.93)	0.76 (0.49–1.18)	1.14 (0.83–2.06)	1.48 (0.71–2.13)	0.005*
Positive TPOAb, n (%)	10 (10.42)	5 (15.63)	2 (6.25)	3 (9.38)	0.764
Positive TRAb, n (%)	2 (2.08)	1 (3.13)	0	1 (3.13)	0.524
Positive TGAb, n (%)	13 (13.54)	7 (21.88)	3 (9.38)	3 (9.38)	0.483
FT3-test delay, median(IQR), days	17 (12.5–28.5)	17 (13.5–27.5)	17 (11.5–30.5)	17.5 (12.5–28)	0.902
**Data obtained during hospitalization**					
Pneumonia, n (%)	54 (56.25)	23 (71.88)	20 (62.50)	11 (34.38)	0.008*
Tumor presence, n (%)	5 (5.21)	2 (6.25)	2 (6.25)	1 (3.12)	1
Abnormal MRI, n (%)	55 (57.29)	18 (56.25)	22 (68.75)	15 (46.88)	0.232
CSF tests					
CSF with pleocytosis, n (%)	68 (70.83)	27 (84.38)	20 (62.5)	21 (65.63)	0.135
CSF WBC count > 20 cells/μL, n (%)	45 (46.88)	18 (56.25)	16 (50)	11 (34.38)	0.192
CSF with oligoclonal bands, n (%)	22 (22.92)	6 (18.75)	11 (34.38)	5 (15.63)	0.182
New symptoms during hospitalization					
Psychiatric behavior, n (%)	76 (79.17)	28 (87.5)	24 (75)	24 (75)	0.398
Seizures, n (%)	63 (65.63)	20 (62.5)	20 (62.5)	23 (71.88)	0.692
Consciousness declination, n (%)	41 (42.71)	21 (65.63)	11 (34.38)	9 (28.13)	0.006*
Central hypoventilation, n (%)	16 (16.67)	11 (34.38)	3 (9.38)	2 (6.25)	0.007*
Immunotherapy					
First-line immunotherapy, n (%)	91 (94.79)	30 (93.75)	31 (96.88)	30 (93.75)	1
Second-line immunotherapy, n (%)	12 (12.5)	3 (9.38)	4 (12.5)	5 (15.62)	0.925
Combined immunotherapy, n (%)	12 (12.5)	3 (9.38)	4 (12.5)	5 (15.62)	0.925
Immunotherapy delay, median (IQR), days	18 (13–31)	18 (14–29)	18 (12–32)	18.5 (13–30)	0.884
Immunotherapy delay > 4 weeks, n (%)	26 (28.57)	8 (26.67)	10 (32.36)	8 (26.67)	0.877
Disease severity					
ICU admission, n (%)	52 (54.17)	28 (87.5)	14 (43.75)	10 (31.25)	< 0.001*
Maximum mRS during hospitalization, median (IQR)	4 (3–5)	5 (4–5)	3 (3–5)	3 (3–3.5)	0.011*
Hospital stay, median (IQR), days	19 (13.5–29)	32 (20–40.5)	18 (13.5–23)	16 (13–20)	< 0.001*
**Data obtained during follow up**					
Lack of clinical improvement within 4 weeks of starting treatment, n (%)	28 (30.77)	16 (53.33)	7 (22.58)	5 (16.67)	0.006*
The NEOS score, mean (SD)	2.18 (1.17)	2.8 (0.96)	2.16 (1.16)	1.57 (1.07)	< 0.001*
Poor one-year outcome (mRS 3–6), n (%)	23 (23.96)	15 (46.88)	5 (15.62)	3 (9.38)	0.002*

IQR, interquartile range; SD, standard deviation; MRI, magnetic resonance imaging; WBC, white blood cell; CRP, c-reactive protein; CSF, cerebrospinal fluid; FT3, free triiodothyronine; FT4, free thyroxine; TSH, thyroid stimulating hormone; TGAb, anti-thyroglobulin antibodies; TPOAb, anti-thyroid peroxidase antibodies; TRAb, anti-thyroid receptor antibodies; ICU, intense care unit; mRS, modified Rankin scale; NEOS, anti-NMDAR Encephalitis One-Year Functional Status.

*P < 0.05.

^#^The new symptoms during hospitalization refer to those firstly manifest after 24 h of admission.

### Comparisons of clinical features among subgroups

Based on the serum FT3 levels, the patients were equally divided into 3 subgroups, including the low-FT3 subgroup (FT3 value < 4.13 pmol/L), the middle-FT3 subgroup (FT3 value 4.13–4.84 pmol/L) and the high-FT3 subgroup (FT3 value > 4.84 pmol/L) (Fig. 1). In view of clinical data obtained within 24 h of admission (Table 1), there were no differences in gender, clinical symptoms, the modified Rankin scale (mRS) score, ICU admission, FT4 levels, FT3-test delay and thyroid antibodies among subgroups. However, compared with those in the high-FT3 subgroup, patients in the low-FT3 subgroup had an older age at onset (34 [23.5–48.5] vs. 26 [17–33] years old, *P* = 0.003) and lower TSH levels (0.76 [0.49–1.18] vs. 1.48 [0.71–2.13] µIU/ml, *P* = 0.001). Besides, their underline infectious or stress status tended to be more common, indicated by higher levels of serum WBC (9.15 [7.45–12.8] vs. 6.99 [6.2–8.9] × 10^9^/L, *P* = 0.049) and c-reactive protein (CRP) (6.87 [2.21–11.11] vs. 1 [0.17–2.2] mg/L, *P* < 0.001). The difference remained significant between the low-FT3 subgroup and the middle-FT3 subgroup for levels of CRP (6.87 [2.21–11.11] vs. 1.69 [0.74–3.02] mg/L, *P* = 0.025).

In Regard of the clinical data obtained during hospitalization (Table 1), there were no differences in tumor presence, abnormal MRI, CSF tests and immunotherapy among subgroups. However, compared with those in the high-FT3 subgroup, patients in the low-FT3 subgroup seemed to be more likely to deteriorate after admission, indicated by higher rates of consciousness declination (65.63% vs. 28.13%, *P* = 0.01) and central hypoventilation (34.38% vs. 6.25%, *P* = 0.036). Their chance of accompanying pneumonia was also higher (71.88% vs. 34.48%, *P* = 0.01). As expected, compared with those in the high-FT3 subgroup, patients in the low-FT3 subgroup had higher disease severity during the acute phase, measured by greater maximum mRS scores during hospitalization (5 [4, 5] vs. 3 [3–3.5], *P* < 0.001), higher percentages of ICU admission (87.5% vs. 31.25%, *P* < 0.001) and longer hospital stay (32 [20–40.5] vs. 16 [13–20], *P* < 0.001). The differences remained significant between the low-FT3 subgroup and the middle-FT3 subgroup for frequencies of consciousness declination (65.63% vs. 34.38%, *P* = 0.042) and ICU admission (87.5% vs. 43.75%, *P* = 0.002), the maximum mRS score during hospitalization (5 [4, 5] vs. 3 [3–5], *P* < 0.001) and the length of hospital stay (32 [20–40.5] vs. 18 [13.50–23], *P* = 0.001).

Since some patients’ length of hospital stay was shorter than 28 days, the data of “lack of clinical improvement within 4 weeks of starting treatment” were fully completed during follow-up visits. Compared with those in the high-FT3 subgroup, patients in the low-FT3 subgroup were more likely to lack clinical improvement within 4 weeks (53.33% vs. 16.67%, *P* = 0.013), had higher NEOS scores (2.8 [0.96] vs. 1.57 [1.07], *P* = 0.001), and were prone to achieve poor 1-year outcome (46.88% vs. 9.38%, *P* = 0.007). The differences remained significant between the low-FT3 subgroup and the middle-FT3 subgroup for lack of clinical improvement within 4 weeks (53.33% vs. 22.58%, *P* = 0.047) and poor 1-year outcome (46.88% vs. 15.62%, *P* = 0.029).

### Association between the FT3 and ICU admission

Considering that patients in the low-FT3 subgroup had significant higher rates of ICU admission, whereas the percentages of ICU admission upon admission were approximately identical among different subgroups, we performed the univariate and multivariate logistic regression analysis to assess the relationship between the FT3 and ICU admission (Table 2). Given the phenomenon that the FT3 level in critical illness might change over time^15,16^, we only analyzed the data obtained within the same period as FT3, i.e. within 24 h of admission. The tumor presence, although discovered later during hospitalization, was also included as it could not change in a short time. The level of FT3 (*P* < 0.001) was significantly correlated with ICU admission in the univariate regression model. Other related factors included consciousness declination (*P* = 0.005), psychiatric behavior (*P* = 0.021), the WBC count (*P* = 0.003) and the TSH (*P* = 0.011). The multivariate regression model confirmed the FT3 as an independent factor (*P* = 0.002) after adjustments for the other 4 confounding factors.

**Table 2:** Univariate and multivariate logistic regression analysis of predictors obtained within 24 h of admission for ICU admission during hospitalization.

Variable	Univariate regression model		Multivariate regression model	
	OR (95% CI)	*P*	OR (95% CI)	*P*
FT3, pmol/L	0.256 (0.129–0.508)	< 0.001*	0.291 (0.134–0.634)	0.002*
Consciousness declination	6.638 (1.795–24.543)	0.005*	7.589 (1.629–35.354)	0.01*
Psychiatric behavior	3.143 (1.189–8.307)	0.021*	2.191 (0.631–7.614)	0.217
WBC count, × 10^9^/L	7.185 (1.947–26.511)	0.003*	3.057 (0.647–14.458)	0.159
TSH, uIU/ml	0.556 (0.353–0.875)	0.011*	0.668 (0.406–1.099)	0.112

In the univariate model, the eligible variables obtained within 24 h of admission and tumor presence were analyzed, and those identified as not predictors of ICU admission during hospitalization (*P* > 0.05) were not shown in this table. In the multivariate regression model, the predictive value of FT3 was adjusted by consciousness declination, psychiatric behavior, WBC count and TSH within 24 h of admission. *Indicates *P* < 0.05.

OR, odds ratio; CI, confidence interval; FT3, free triiodothyronine; WBC, white blood cell; ICU, intense care unit; TSH, thyroid stimulating hormone.

### Correlation between the FT3 and 1-year outcome

In the univariate regression model assessing all clinical data obtained within 4 weeks of starting treatment (Table 3), the level of FT3 was significantly correlated with 1-year outcome (*P* = 0.004). Other related factors included gender (*P* = 0.008), age (*P* = 0.034), tumor presence (*P* = 0.018), consciousness declination (*P* = 0.047), central hypoventilation (*P* = 0.002), ICU admission (*P* = 0.003), lack of clinical improvement within 4 weeks (*P* < 0.001) and the NEOS score (*P* < 0.001). However, the prognostic value of the FT3 was attenuated (*P* = 0.594) after adjustment for other confounding factors. The NEOS score turned out to be the sole predictor independently related to poor 1-year outcome (*P* = 0.007).

**Table 3:** Univariate and multivariate analysis of predictors obtained within 4 weeks of starting treatment for poor one-year outcome.

Variable	Univariate regression model		Multivariate regression model	
	OR (95% CI)	*P*	OR (95% CI)	*P*
FT3, pmol/L	0.385 (0.202–0.732)	0.004*	0.792 (0.337–1.863)	0.594
Male	0.267 (0.094–0.703)	0.008*	0.371 (0.106–1.297)	0.120
Age, years	3.457 (1.101–10.851)	0.034*	1.512 (0.294–7.764)	0.620
Tumor presence	15.158 (1.599–143.649)	0.018*	8.393 (0.638–110.452)	0.106
Consciousness declination	2.65 (1.012–6.941)	0.047*	0.771 (0.171–3.474)	0.735
Central hypoventilation	6.061 (1.931–19.024)	0.002*	3.120 (0.551–17.685)	0.199
The NEOS score	3.337 (1.843–6.041)	< 0.001*	2.674 (1.301–5.495)	0.007*
ICU admission	5.758 (1.782–18.599)	0.003*	–	–
Lack of improvement within 4 weeks of starting treatment	36 (8.935–145.053)	<0.001*	–	–

In the univariate model, the eligible variables obtained within 4 weeks of starting treatment were analyzed and those identified as not predictors of poor one-year outcome (*P* > 0.05) were not shown in this table. In the multivariate regression model, the predictive value of FT3 was adjusted by gender, age, consciousness declination, tumor presence, central hypoventilation and the NEOS score.

OR, odds ratio; CI, confidence interval; FT3, free triiodothyronine; ICU, intense care unit; NEOS, anti-NMDAR Encephalitis One-Year Functional Status.

Considering the fact that the level of FT3 might change over time in critical illness^15,16^, the clinical data obtained within the same period as the FT3 would be more comparable and suitable to assess the role of FT3. Therefore, we performed another univariate logistic regression analysis of the clinical data obtained within 24 h of admission and the tumor presence (Table 4), 5 factors turned out to be significant, including the FT3 (*P* = 0.004), gender (*P* = 0.008), age (*P* = 0.034), tumor presence (*P* = 0.018) and central hypoventilation upon admission (*P* = 0.01). The prognostic value of the FT3 remained to be significant (*P* = 0.030) after adjustment for the other 4 confounding factors.

**Table 4:** Univariate and multivariate logistic regression analysis of predictors obtained within 24 h of admission for poor one-year outcome.

Variable	Univariate regression model		Multivariate regression model	
	OR (95% CI)	*P*	OR (95% CI)	*P*
FT3, pmol/L	0.385 (0.202–0.732)	0.004*	0.437 (0.207–0.923)	0.030*
Male	0.267 (0.094–0.703)	0.008*	0.265 (0.001–0.869)	0.028*
Age, years	3.457 (1.101–10.851)	0.034*	2.849 (0.658–12.327)	0.161
Tumor presence	15.158 (1.599–143.649)	0.018*	11.363 (1.071–120.566)	0.044*
Central hypoventilation	6.088 (1.544–24.006)	0.01*	6.833 (1.442–32.382)	0.015*

In the univariate model, the eligible variables obtained within 24 h of admission and the tumor presence were analyzed, and those identified as not predictors of poor one-year outcome (*P* > 0.05) were not shown in this table. In the multivariate regression model, the predictive value of FT3 was adjusted by gender, age, the tumor presence, and central hypoventilation within 24 h of admission. *Indicates *P* < 0.05.

OR, odds ratio; CI, confidence interval; FT3, free triiodothyronine.

## Discussion

This study has demonstrated two major findings. First, the patients with relatively low FT3 within 24 h of admission tend to have higher disease severity during hospitalization and worse outcome in follow-up visits, represented by higher chances of ICU admission, longer hospital stay, greater maximum mRS scores during hospitalization, lower rates of getting clinical improvement within 4 weeks of starting treatment, and higher percentages of achieving poor one-year outcome. Second, the level of FT3 was an independent factor correlated with ICU admission during hospitalization and might serve as a potential predictor for 1-year outcome. Taken altogether, our results reveal that the FT3 might be a risk factor involved in the evolution and progression of anti-NMDAR encephalitis.

Thyroid hormones are able to cross the blood–brain barrier and affect neurogenesis, cell differentiation, and myelination^17,18^. A growing body of literature shows that the FT3 is also closely involved in regulating neutrosphere biology and immune system function (e.g., cell-mediated immunity)^19,20^. Anti-NMADR encephalitis is mediated by antibodies to neuronal surface antigens, with 25% suffer significant morbidity or mortality^21,22^. Given the critical role of the FT3 in the proliferation and differentiation of neuronal and glial progenitors during brain development and in the regulation of immune system function^17,23^, we hypothesize that the FT3 might be a risk factor in the progression and outcome in anti-NMDAR encephalitis. The preliminary results of the study bolstered this assumption, because: (1) Patients in the low-FT3 subgroup, although with the similar symptoms and mRS scores upon admission compared to those in the high-FT3 subgroup, seemed to be more likely to aggravate during hospitalization and exhibit higher disease severity, indicated by greater maximum mRS scores during hospitalization, longer hospital stay, and higher rates of ICU admission. After adjustment for relevant clinical variables, the level of FT3 turned out to be an independent prognostic factor related to ICU admission; (2) Patients in the low-FT3 subgroup had much higher chances of achieving poor one-year outcome, and the univariate regression analysis confirmed the level of FT3 as a significant prognostic factor, although its predictive value was attenuated after adjustment for other confounding factors obtained within 4 weeks of starting treatment. However, since the level of FT3 might change over time in critical illness^15,16^, the clinical data obtained within the same period as the FT3 would be more comparable and suitable to assess the role of FT3. Therefore, we performed another multivariate regression analysis of clinical data obtained within 24 h of admission. The tumor presence, although discovered later during hospitalization, was also analyzed as it could not change in a short time. As expected, the predictive value of the FT3 remained significant after adjustment for gender, age, the tumor presence and central hypoventilation obtained within 24 h of admission.

The mechanisms of how the FT3 influences the evolution and outcome of anti-NMDAR encephalitis remain elusive. First, we assume that patients with low FT3 might experience decreased neuroprotection and increased secondary brain damage after anti-NMDAR encephalitis, leading to higher disease severity during hospitalization and poorer outcomes. In some vitro studies, the FT3 has been proven to exert a protective effect against glutamate toxicity in neurons and glial cells through both transcriptional and non-transcriptional mechanisms^24,25^. Second, patients with low FT3 might exhibit a suppression of endogenous brain repair systems in light of the crucial role of the FT3 in the generation and maturation of new neurons and axonal myelination^26^. Increasing evidence suggests that the FT3 is closely related to brain acetylcholine activity, cholinergic function, and the secretion of various neurotrophic factors such as nerve growth factor, which are pivotal to the brain repair system^27^. However, since several critical conditions may have decreased FT3 levels^6,7,10^, and the exact immunologic relevance of FT3 in anti-NMDAR encephalitis remains obscure^19,20^, the FT3 might as well be at first interpreted as a biological risk factor, but not as a causal factor directly altering outcome of patients with anti-NMDAR encephalitis.

In the multivariate regression model assessing all clinical data obtained within 4 weeks of starting treatment, the predictive value of the NEOS score was validated in this prospective cohort, which provided another Class III evidence of using the NEOS score as a tool to predict 1-year functional status in patients with anti-NMDAR encephalitis^3,5^. It is of interest to notice that patients in the low-FT3 subgroup had higher NEOS scores, which might provide a clue to predict the NEOS score in the early stage of the disease.

Several limitations merit consideration in the interpretation of this study. First, it is observational in nature and based on data from a single hospital, which might have led to unintentional bias. Second, the number of patients in this analysis is of only moderate size limiting the statistical power. Third, we only evaluated serum thyroid hormones at a single time point. The level of FT3 in critical illness changes over time, which is a dynamic process^15,16^. Future studies evaluating multiple time points are required for validating the predictive role of FT3 in anti-NMDAR encephalitis.

In conclusion, our analysis reveals that the FT3 might be a risk factor involved in the evolution and progression of anti-NMDAR encephalitis, whereas the underline mechanisms remain to be explored. Attention should be paid to these patients with relatively low FT3 upon admission, which might possibly aid clinical prediction and guide clinical decision-making. Further investigations in larger cohort of patients are definitely needed to confirm the prognostic value of FT3.

## Methods

### Subjects and evaluation

In this prospective study, we evaluated consecutive inpatients with anti-NMDAR encephalitis admitted in the Zhengzhou University First Affiliated Hospital between January 1, 2016 and December 31, 2019. Depending on the clinical status, informed consent was obtained from the patients or their relatives. Prior approval of the study protocol was obtained from the ethical committee. All patients’ records and information were anonymized and de-identified prior to analysis. The inclusion criteria were (1) met the diagnostic criteria of definite anti-NMDAR encephalitis, i.e. in the presence of one or more of the six major groups of symptoms and positive IgG anti-GluN1 antibodies in the CSF, after reasonable exclusion of other disorders^1^; (2) thyroid hormones obtained within 24 h of admission, including serum FT3, FT4 and TSH.

Follow-up visits started from the day of diagnosis, i.e. the day of obtaining positive IgG anti-GluN1 antibodies in the CSF. Each patient underwent a follow-up evaluation by telephone or outpatient interview for 12 months. The mRS values during hospitalization were calculated by referring physicians and medical records. A favorable outcome was defined as a mRS score of 0–2 and a poor outcome was defined as a score of 3–5 or death (mRS score of 6).

### Clinical information and thyroid function measurements

Clinical data and physical examination were obtained in all patients upon admission. Blood samples were collected within 24 h of admission and, in addition to standard blood tests, serum levels of FT3, FT4 and TSH were determined using a direct chemiluminescence assay (ADVIA, Bayer Health Care LLC Tarrytown, NY, USA). The reference intervals of our laboratory were as follows: 3.28–6.47 pmol/L for FT3, 7.9–18.4 pmol/L for FT4, and 0.34–5.6 µIU/ml for TSH. A small number of patients also received tests of thyroid autoantibodies, including anti-thyroglobulin antibodies (TGAb), anti-thyroid peroxidase antibodies (TPOAb), and anti-thyroid receptor antibodies (TRAb)^13,14^. “Clinical hyperthyroidism” was defined as suppressed TSH and elevated FT4 levels with relevant clinical symptoms, and “subclinical hyperthyroidism” was defined as a suppressed TSH level, FT3 and FT4 levels within the normal ranges, and the absence of symptoms. In contrast, “clinical hypothyroidism” was defined as elevated TSH and suppressed FT4 levels with relevant symptoms, and “subclinical hypothyroidism” was defined as an elevated TSH level, a normal FT4 level, and the absence of symptoms. “Low T3 syndrome” was defined as a low serum FT3, normal–low FT4, and normal–low TSH levels^28^. “Other conditions” included slightly elevated FT3 or FT4 levels, normal TSH and the absence of symptoms.

During hospitalization, all patients underwent the lumbar puncture, and the IgG anti-GluN1 antibodies in the CSF were detected by cell-based assays (CBA), using Euroimmun IIFT kits: Autoimmune Encephalitis Mosaic 1 (FA 1121-1005-1), and/or NMDAR kits (FA112d-1005-51), according to the manufacturer’s instructions^1^. All measurements were performed by laboratory staff who were blinded to patients’ clinical information. All patients received the following examinations to screen tumors during hospitalization, including the brain MRI, computerized tomography (CT) scan of the thorax, ultrasound of the abdomen, pelvic and reproductive regions. None of the patients were given methylprednisolone or any other immunotherapies before admission, and the immunotherapies were not started until the GluN1 antibodies in the CSF were confirmed.

The “pneumonia” was diagnosed by respiratory physicians according to the relevant criteria. The “tumor presence” was defined as having ovarian teratoma or any other malignant tumors. The “abnormal MRI” was defined as having relevant brain lesions confirmed by radiologists. The “FT3-test delay” was defined as the interval between the onset of symptoms and the test of FT3 value upon admission. The “immunotherapy delay” was defined as the interval between the onset of symptoms and the initiation of immunotherapy. The “First-line immunotherapy” included steroids, intravenous immunoglobulins or plasma exchange alone or combined. The “Second-line immunotherapy” comprised rituximab, azathioprine or cyclophosphamide treatment alone or combined^22^. The “Combined immunotherapy” was defined as having both first-line and second-line immunotherapies. The “NEOS score” contained 5 variables including ICU admission, treatment delay > 4 weeks, lack of clinical improvement within 4 weeks of starting treatment, abnormal MRI, and CSF white blood cell (WBC) count > 20 cells/μL, each of which was assigned 1 point.

### Statistical analysis

The categorical variables were expressed as counts (%), whereas the continuous variables were expressed as the mean (standard deviation, SD) or median (interquartile range [IQR]) values as appropriate. The age, immunotherapy delay, hospital stay, WBC and the concentrations of CRP were log-transformed to approximate a normal distribution. The univariate logistic regression was used to identify factors significantly associated with an increased risk of poor outcome; for each variable, the odds ratio (OR), and 95% confidence interval (CI) were given. Relevant variables with *P* < 0.05 in the univariate logistic regression analysis were entered into multivariate logistic regression models to identify whether variables were independently associated with outcome.

Patients were equally divided into 3 subgroups based on their serum FT3 levels. Baseline demographic and clinical features were compared across the three subgroups using the one-way analysis of variance (ANOVA), the equality-of-medians test or the Fisher’s exact test as appropriate. For differences within subgroups, the pair wise comparison with Bonferroni correction was used. All analyses were performed using Stata for Windows, Version 14.0 (StataCorp LLC., USA). *P* value < 0.05 was considered to be statistically significant.

### Ethical approval

The Ethics Committee of the Zhengzhou University First Affiliated Hospital approved this study. This study was performed in accordance with the 1964 Declaration of Helsinki and later amendments.

### Consent to participate and publication

Written informed consents were obtained from all patients or their guardians prior to their inclusion for sample collection and publication of this paper.

## Data Availability

Data that support the findings of this study are available upon reasonable request.
